# Green Synthesis and Anti-Inflammatory Activity of Silver Nanoparticles Based on Leaves Extract of *Aphania senegalensis*

**DOI:** 10.1155/2024/3468868

**Published:** 2024-09-19

**Authors:** Fatemata Diallo, Insa Seck, Samba Fama Ndoye, Tamsir Niang, Sidy Mouhamed Dieng, Fatou Thiam, Moussa Ndao, El Hadji Mamour Sakho, Alioune Fall, Madièye Séne, Matar Seck

**Affiliations:** ^1^ Laboratoire de Chimie de Coordination Organique et Bioorganique (LCCOB) Université Cheikh Anta DIOP de Dakar (UCAD), BP 5005 Dakar-Fann, Dakar, Senegal; ^2^ Laboratoire de Chimie Organique Chimie Physique et Chimie Thérapeutique Université Cheikh Anta DIOP de Dakar (UCAD), BP 5005 Dakar-Fann, Dakar, Senegal; ^3^ Institut de Chimie et des Matériaux Paris-Est (ICMPE) 94320, Thiais-France, Paris, France; ^4^ Laboratoire de Pharmacie Galénique Département de Pharmacie Université Cheikh Anta DIOP de Dakar (UCAD), BP 5005 Dakar-Fann, Dakar, Senegal; ^5^ Faculté des Sciences et Techniques Université Cheikh Anta Diop de Dakar (UCAD), BP 5005 Dakar-Fann, Dakar, Senegal; ^6^ Laboratoire de Pharmacologie Département de Pharmacie Université Cheikh Anta DIOP de Dakar (UCAD), BP 5005 Dakar-Fann, Dakar, Senegal

## Abstract

This study focuses on the synthesis of silver nanoparticles (AgNPs) using the extract of *Aphania senegalensis* leaves. The extraction was done using maceration at room temperature in water for 48 h. The synthesized nanoparticles were characterized by IR, XRD, TEM, and SEM. The thermal stability of these nanoparticles was studied by TGA. The zeta potential was used to define the size, charge distribution, and stability of the nanoparticles. Optimization reactions were carried out based on reaction time, pH, and temperature. The nanoparticles obtained from optimal conditions were evaluated on induced inflammation. The determination of the average diameters and geometry of nanoparticles was carried out by XRD by calculating the lattice constants, and they are between 18.11 and 50 nm. The evaluation of anti-inflammatory activity showed that the nanoparticles are 10 times more active than the extract of *Aphania senegalensis* leaves. Minimum doses of 10 mg/kg orally and 3 mg/kg were obtained for the plant extract, respectively. These results are promising for the possibility of AgNPs to be used for the treatment of inflammation.

## 1. Introduction

Nanotechnology is considered one of the most important technologies due to the small size of the materials involved, known as nanomaterials, and their wide range of applications [1, 2]. Nanomaterials have diameters between 1 and 100 nm. This variation in sizes depends on the nature, source, or method of obtaining the material. There are several methods for synthesizing nanoparticles. We can cite chemical, physical, and biological methods. Some are very dangerous for environment, while others are green, in this case the use of plant extracts or microorganisms. These depend on the synthesis method. For green synthesis with extracts, we can target biological properties as an area of application since most of these plants are used in traditional medicine. Silver nanoparticles can be synthesized using various methods, but green synthesis is often considered the best method. Indeed, nanomedicine is a current field and silver nanoparticles are widely used as drug mediators and in imaging due to their small sizes and their contact surface [3]. The interest in green synthesis with extracts is because the latter are full of molecules which have oxidative properties, and which also play the role of stabilizers. This green method is also very economical, ecological, and easily accessible [4, 5]. Many plants like *Citrus macroptera* [6], *Catharanthus roseus* and *Ocimum tenuiflorum* [7], *Citrus sinensis*, *Solanum tricobatum*, *Centella asiatica*, *Syzygium cumini* [8], and *Terminalia mantaly* [9] are used to synthesize silver nanoparticles. Depending on the part of the plant used, silver nanoparticles with good properties can be used. Secondary metabolites are responsible of bioactivity of plant extracts and involve biogenic metal nanoparticles synthesis. The areas of application of silver nanoparticles as antimicrobials, anti-inflammatory, electronic materials, and electronic tools depending on their properties [10, 11]. Previous work demonstrated that bio AgNPs contribute to the treatment of infections or chronic inflammation [12, 13]. Inflammation is a response of the immune system to fight infections. Many diseases such as cancer, rheumatoid arthritis, asthma, and diabetes are often accompanied by chronic infections or uncontrolled inflammations. TNF-*α*, IL-6, and IL-1*β* promote inflammation but excessive or uncontrolled production of these cytokines can develop chronic obstructive pulmonary disorder, osteoarthritis, and cancer [14].

In this work, we focus on the silver nanoparticles synthesis using *Aphania senegalensis* (*A. senegalensis*) leaves extract and their anti-inflammatory activity. *A. senegalensis* is a well-known plant in Senegal and Africa. *A. senegalensis* belongs to the Sapindaceae family, which includes dicotyledonous plants. The leaves of *A. senegalensis* possess antioxidant [15], antimicrobial [16], cytotoxic [16, 17], and anti-inflammatory and pain-killing [18]. The *A. senegalensis* leaf contains various active compounds such as flavonoids, tannins, steroids, and triterpenes [15, 16, 19]. The leaf extract contains 151.63 mg tannic acid/g [20]. These compounds serving as antioxidants can reduce Ag^+^ to Ag^0^ and realize the synthesis of AgNPs in large-scale production. The parameters of AgNPs synthesis such as time, pH, and temperature were studied to optimize the synthesis of AgNPs from *A. senegalensis* leaves extract. Ultraviolet-visible (UV-vis) spectroscopy, Fourier transform infrared (FTIR), X-ray diffraction (XRD), thermogravimetry analysis (TGA), potential measurement, and dynamic light scattering (DLS) were used to characterize AgNPs. Results obtained from this study could be the first step for the discovery of new anti-inflammatory drugs. The AgNPs were never synthesized by *Aphania senegalensis* leaves extract and from the leaves extract to the AgNPs we noted that the anti-inflammatory activity was improved.

## 2. Materials and Methods

### 2.1. Preparation of the Plant Extract

The fresh leaves were harvested on July 3, 2023, at 7 : 30 am. in a village called Ndjender located 50 km northeast of Dakar. The plant was identified and deposited under voucher specimens numbered K1613 at the herbarium of the botanical garden of this faculty. Leaves were washed and dried away from light for a week. The extraction was carried out by cold maceration for 48 hours. 100 g of powder were impregnated in 400 mL of water and the mixture is kept stirring with a magnetic stirrer. After 48 hours the mixture is filtered, and the solvent renewed for a second 48 hours. The filtrates recovered were evaporated to dryness using a rotary evaporator with a temperature of 40°C. and a rotation of 250 rotations/minute. The extract was stored at 4°C.

### 2.2. Materials

AgNO_3_ and NaOH were purchased from Oxford Lab Fine Chemistry L.

All the reagents were used without further purification.

**Measurements**. UV-vis spectroscopic analysis was performed on JASCO spectrometers. Quartz cuvettes were used. The hydrodynamic size and zeta potential were measured through DLS analysis performed on a Mastersizer 3000 (Malvern Panalytical) Zetasizer Nano ZS instrument. FTIR spectra were recorded on a JASCO, FT/IR-460 plus spectrometer using KBr pellets with a scan of 4 cm^−1^s^−1^ approximately at 25 °C. XRD analysis was carried out on a BRUKER D8 DA VINCI diffractometer with the LYNXEYE quick detector. TEM (PHILIPS CM200).

### 2.3. Biogenic Synthesis of AgNPs

The AgNPs were obtained based on Leena's method with slight modifications [21]. The extract of *Aphania senegalensis* was dissolved in 25 mL of water and a fresh solution (58.8 mM, 100 mL) of silver nitrate (AgNO_3_) was prepared. The latter in a 250 mL flask is stirred. The extract solution is added gradually in portions into the silver nitrate solution. The reaction was at 70°C for 2 hours. During the reaction, the pH of the reaction medium was adjusted with a NaOH solution (2 g in 100 mL). A color change from dark brown to orange yellow was noted. The nanoparticles were washed by centrifugation at 10,000 rpm for 30 min, followed by washing with distilled water to remove impurities.

### 2.4. Characterization of the NPs

The characterization is an important step which allows not only to prove the formation of nanoparticles but also to understand their properties, their behaviors, and possibly their areas of application. XRD was used to characterize the crystallinity, geometry, and purity of the nanoparticles. A PanAlytical X'Pert Pro RX diffractometer was used with a diffraction angle of 2 theta from 20° to 90° with a scanning speed of 20°/min. Infrared has also been used to characterize the functional groups present in nanoparticles. The PerkinElmer, UV-vis Lamba 365 apparatus was used with a wavenumber interval ranging from 4000 cm to 450 cm. The MET JEAOL 200 EX brand transmission electron microscope (TEM) was used to analyze the size and shape of the nanoparticles.

#### 2.4.1. Potential Measurements

The zeta potential measurements were performed by laser Doppler anemometry with a Nano-ZS zetasizer (Malvern Instrument).

#### 2.4.2. Dynamic Light Scattering (DLS)

The distribution of suspended particles in the solution was determined by Dynamic Light Scattering (DLS). This technique is often also used for nanoscale particles to analyze their polydispersity and sizes. Instrument brand device (Malvern) with a wavelength of 633 nm and a scattering angle of 90° was used.

### 2.5. Anti-Inflammatory Activity

Anti-inflammatory activity was evaluated following inflammation caused by carrageenan using rats. This study was validated by the ethical committee of research (CER) of Cheikh Anta Diop University under the approval number 0372/2023/CER/UCAD. The rats were divided into three groups (Table 1) and fasted for 12 hours before testing. The initial diameter of the left hind paw (D0) was measured using a digital caliper. Injection of 1% carrageenan solution (1 ml) into the planter region of the left hind paw of rats induced rat paw oedema after oral administration with different solutions. The increased edema was measured using a digital caliper at 60, 180, and 300 min (T1 h, T3 h, and T5 h) after carrageenan injection. The importance of edema was evaluated by the mean percentage of increasing diameter of the rat paw according to the following formula:

(1) $$\begin{array}{c} \%\text{INC}=\frac{\text{Dt}-D0}{D0}\times 100, \end{array}$$

where Dt: paw diameter at *t* time; D0: initial paw diameter.

## 3. Results and Discussion

**Figure 1:**
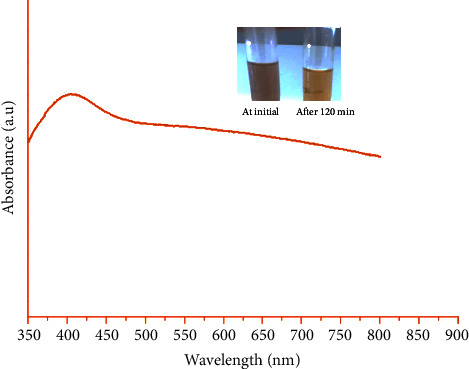
UV-vis spectrum of AgNPs.

The synthesized silver nanoparticles were first characterized by a color change from brown to yellow noted during the synthesis (Figure 1). This color change was described by Leena et al. [21]. The same phenomenon was observed by Valko after just 60 min of incubation [22]. The plasmonic peak was observed at 405 nm. This proves the formation of silver nanoparticles which give a plasmonic peak varying between 380 nm and 450 nm depending on the size and shape of the nanoparticles [23]. These results show that the leaves of *Aphania senegalensis* are full of potential metal reducing agents.

### 3.1. Effect of the Reaction Time for the Synthesis of AgNPs

**Figure 2:**
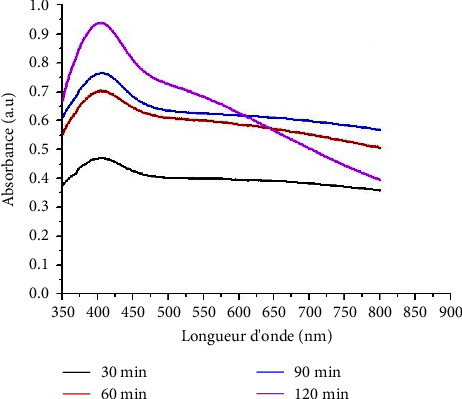
Effect of reaction time on the absorption spectra of the reaction.

The formation of silver nanoparticles was evaluated as a function of time.  Figure 2 shows a strong increase in the concentration of nanoparticles in the reaction medium. This shows that the formation of nanoparticles depends on time. The same phenomenon has been demonstrated in previous works [24, 25]. We see that the plasmonic peak does not change position as a function of time. Which means that over time we have a good homogeneity of the nanoparticles because the position of the peak depends on the size and shape of the nanoparticles [26].

### 3.2. pH Effect on AgNPs Synthesis

**Figure 3:**
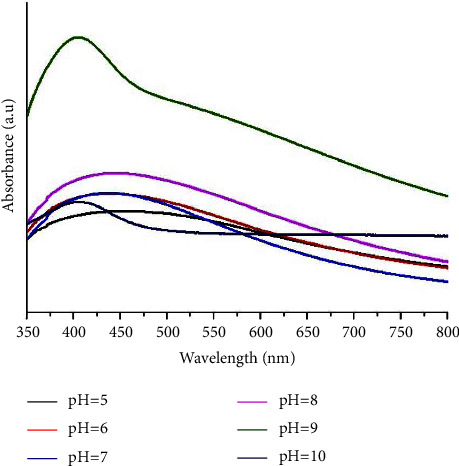
Effect of pH on the absorption spectra of the reaction.

In formation of silver nanoparticles, the influence of pH was sought. It was noted that the formation of nanoparticles is strongly influenced by the pH of the medium. The effects may be on kinetics, stability, and the particles size distribution. These effects were optimized with pH values of 5, 6, 7, 8, 9, and 10 (Figure 3). A slight shift from 405 nm to 425 nm of the plasmonic peak was observed with pH 8 and 9. This phenomenon confirms the formation of nanoparticles which optimal in basic media compared to acidic and neutral media [27]. In addition to the shift of the peak, we also note an increase of the concentration of nanoparticles in the basic medium. This means that the bioreduction of Ag^+^ to Ag^0^ is much easier in an alkaline environment. Indeed, in a basic environment the functional groups engage in the metal reduction reaction. In an acidic environment, the reduction in metal reduction is due to the denaturation and degradation of the bioactive compounds present in the leaf extract [28].

### 3.3. Effect of Temperature for the Synthesis of AgNPs

**Figure 4:**
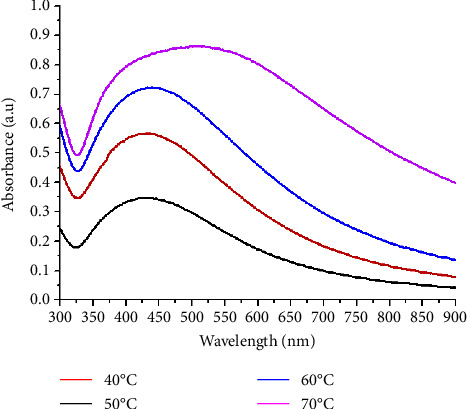
Effect of temperature on the absorption spectra of the reaction.

UV-vis spectra obtained were recorded at 40°C, 50°C, 60°C, and 70°C by keeping the other parameters constant (Figure 4). The presence of a plasmon peak for all samples indicates the formation of AgNPs. The maximum absorbance was recorded at 70°C and the absorption intensity increased with temperature. It means that the formation of silver nanoparticles is depended on the temperature. The bathochromic effect was noted at 60°C and 70°C. This phenomenon means that the diameters of the silver nanoparticles have decreased [29]. The formation of nanoparticles at 40°C indicated the ability of *Aphania senegalensis* leaves extract to reduce the Ag^+^ to Ag^0^ without high temperature but which remains the worst temperature. Contrary to the literature which indicated that the best condition for the synthesis of AgNPs using *Hagenia abyssinica* is at 40°C [30].

### 3.4. Characterization of the Synthesized AgNPs

#### 3.4.1. FTIR Analysis

**Figure 5:**
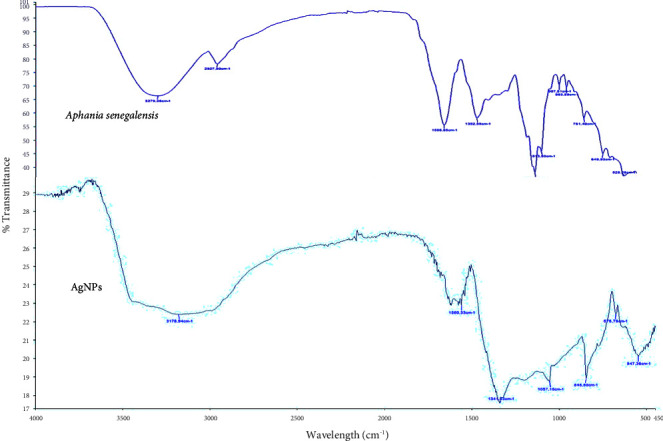
FTIR spectra of *A. senegalensis* extract and AgNPs synthesized.

FTIR is used to analyze the chemical composition and functional groups present on the surface of nanoparticles. It provides information about molecular vibrations and can be used to study surface modifications, coatings, and interactions with other molecules. The FTIR spectrum of the extract and AgNPs are represented in Figure 5. The broad bands of stretching vibrations of OH and NH were observed at 3178 cm ^−1^. A broad shoulder was observed in the lower region, which showed the construction of hydrogen bonds through O–H and N–H bonds within the capping organic molecules on the AgNPs' surface. The comparison between the leaf extract and the AgNPs spectra shows a change in the main characteristic bands of the extract. We note absence of band at 1013 cm^−1^ corresponding to an elongation vibration of the C–O bond. This can be explained by the formation of a dative bond between the free oxygen doublet and the empty orbital of Ag^+^. The peaks at 1560 cm^−1^, 1341 cm^−1^, 1057 cm^−1^ were attributed to the stretching of N–H, C–H of an aromatic ring, and C–O of a carboxylic acid, respectively. The decrease of the OH band from the extract spectra to the AgNPs spectra is due to hydrogen bonds during the metallic silver reduction into nanoparticles. In fact, polyphenolic compounds are attached to the AgNPs to reduce the Ag^0^ and stabilize the AgNPs.

#### 3.4.2. XRD Analysis

**Figure 6:**
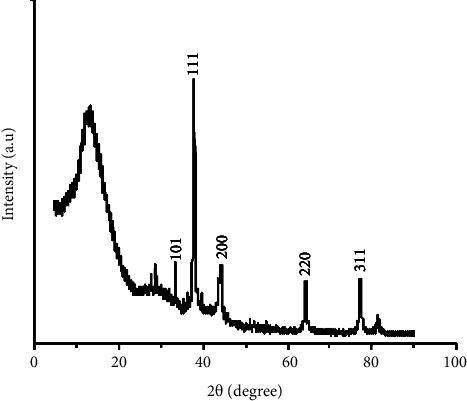
AgNPs XRD spectra of AgNPs synthetized.

XRD analysis provides the crystal structure of silver nanoparticles. AgNPs exhibit a face-centered cubic crystal structure. The values of 2*θ* were recorded between 20° and 80° (Figure 6). The predominant peak is the diffraction peak (111). This peak reveals the pure crystalline silver nitrate of synthesized AgNPs. Different diffraction peaks were observed at 38, 45, 65, and 78, corresponding to the plans (111), (200), (220), and (311), respectively [7]. These plans confirmed that the AgNPs have face-centered cubic structures. The lattice constant values corresponding to these angles/planes are 4.0010, 4.0300, 4.0588, and 4.0680, respectively. The XRD patterns recorded at 29.5° and 82° correspond to the crystalline structure of Ag_2_O_3_. These peaks confirmed the formation of pure AgNPs and Ag_2_O_3_. The average crystalline size using the Scherrer equation was found to be 18.11 nm and 50 nm for synthetized AgNPs.

Scherrer equation:

(2) $$\begin{array}{c} D=\frac{0.94\lambda }{\beta \;\cos\left(\theta \right)}, \end{array}$$

where *D* = average crystalline size, *β* = line broadening in radians, *θ* = Bragg's angle, *λ* = X-ray wavelength (=1.5418).

**Figure 7:**
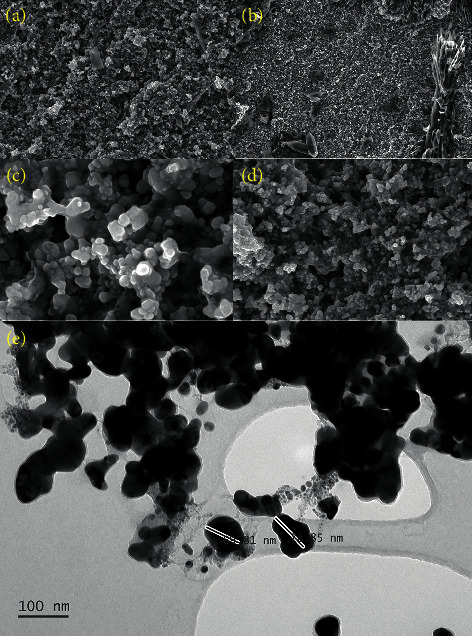
SEM micrograph of synthesized AgNPs at different scales (a) 1 *μ*m, (b) 10 *μ*m, (c) 10 nm, (d) 200 nm, and (e) TEM imaging of synthesized AgNPs scale 100 nm.

The morphology of AgNPs was visualized using SEM and TEM in scale 100 nm (Figure 7).

#### 3.4.3. DLS Analysis of AgNPs

**Figure 8:**
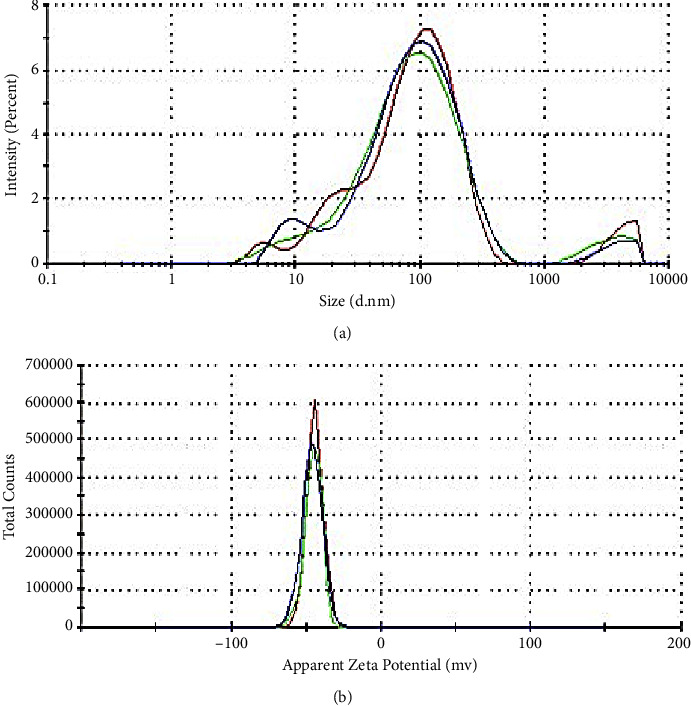
(a) Size distribution and (b) zeta potential distribution of AgNPs synthetized.

DLS method commonly called DLS using zeta size and zeta potential provide the particle size distribution and surface charge of AgNPs. The results show good polydispersity index of 0.697 of AgNPs with an average diameter of 61.8 nm Figure 10 (Figure 8). Zeta potential analysis measures the electrokinetic potential of nanoparticles in solution, providing insights into their surface charge and stability. The zeta potential of −45 mV proves that the capping organic molecules on the surface of AgNPs are negatively charged. It should also be noted that this low value confirms the stability of AgNPs. Indeed, with the lower values of the zeta potential, more synthesized nanoparticles are stable. This relatively negative value provides an excellent stability of AgNPs due to electrostatic repulsion between particles. Hydroxylate groups within polyphenolic compounds are the source of negative zeta potential. Complete dispersion stability by electrostatic repulsion requires a zeta potential of >+30 or <−30 mV [31, 32]. Various phytochemicals including polyphenols have a high degree of stability of green synthesized AgNPs and could be achieved with zeta potential values not in abovementioned range [32, 33].

#### 3.4.4. DSC/TGA Analyses

**Figure 9:**
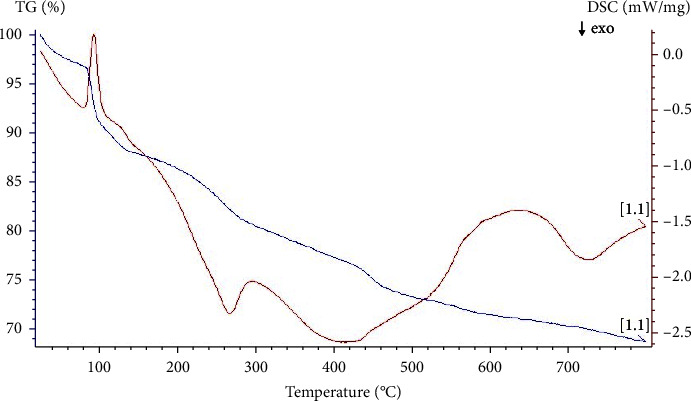
Simultaneous DSC/TGA of synthesized AgNPs analysis.

Differential Scanning Calorimetry (DSC) and Thermogravimetric Analysis (TGA) analyze the thermal properties and stability of nanoparticles, including melting points, phase transitions, and decomposition temperatures. TGA and DSC analysis were performed on synthesized AgNPs using argon. The TGA plot (Figure 9) showed a weight loss in the temperature range of 100–800°C. The weight loss of AgNPs due to the desorption of capping bioorganic compounds on the AgNPs surface [34], and the impurities vaporization was 31%. At the highest temperature of 800°C, the nature of AgNPs is altered and cannot be used in high-temperature consuming reactions.

### 3.5. Anti-Inflammatory Activity

**Figure 10:**
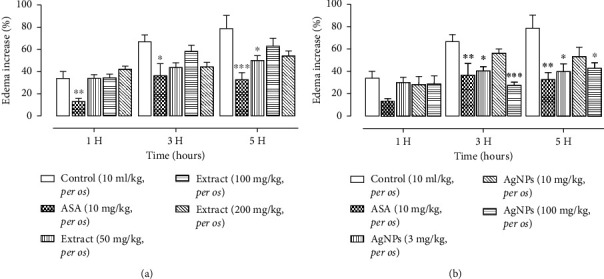
(a) Extract of *A. senegalensis* and (b) AgNPs synthesized effects on inhibition of edema.

Inflammation is the injury of tissue or organ resulting in symptomatic pain, swelling, temperature, and redness. The anti-inflammatory assay revealed that the maximum inhibition (%) of the extract was obtained. *A. senegalensis* extract administered orally prevents the development of inflammatory oedema of the rat paw induced by carrageenan. The effect observed was more pronounced at a dose of 50 mg/kg between 3 and 5 hours after induction of inflammatory oedema (Figure 10). This activity is less significant than that of aspirin. In the same model, AgNPs significantly prevented inflammatory oedema at a lower dose of 3 mg/kg, following the same profile (3 h, 5 h). The activity observed was like that of aspirin used at a higher level. The anti-inflammatory activity extract and AgNPs were found with a minimum dose of 10 mg/kg *per os* and 3 mg/kg *per os,* respectively.

The diverse secondary metabolites of *A. senegalensis* are reported as polyphenols such as flavonoids and tannins (hydrolyzed and condensed) and saponins [35]. These phytochemical groups are known for their antimicrobial [36], antiparasitical, analgesic, and anti-inflammatory activities [18]. In this study, the results showed that the extract obtained from *A. senegalensis* leaves exerted a weak anti-inflammatory effect at the administered dose of 50 mg/kg. However, AgNPs inhibited the rate of inflammatory edema which was 40% at 3 h with 3 mg/kg corresponding to 1 mg/mL, *per day* after injection of carrageenan. The effect does not increase at 5 h. AgNPs have been shown to reduce the production and release of proinflammatory cytokines, such as tumor necrosis factor-alpha (TNF-alpha), interleukin-6 (IL-6), and interleukin-1 beta (IL-1 beta), which play crucial roles in inflammatory responses [37]. AgNPs have been reported to inhibit the activation of nuclear factor-kappa B (NF-kB), a transcription factor that regulates the expression of various genes involved in inflammation and immune responses. By suppressing NF-KB activation, AgNPs can mitigate the inflammatory process [38]. They may modulate immune responses by affecting the function of immune cells such as macrophages, neutrophils, and dendritic cells [39]. These nanoparticles influence the production of reactive oxygen species (ROS) and nitric oxide (NO), which are key mediators of inflammation [40]. In addition to their anti-inflammatory effects, AgNPs possess wound healing properties. By reducing inflammation and promoting tissue regeneration, AgNPs can accelerate the healing process in various types of wounds [41]. It is important to note that while AgNPs show promise as potential anti-inflammatory agents, their use raises concerns regarding safety, including potential toxicity and environmental impact. Further research is needed to elucidate the mechanisms of action, optimize dosing regimens, and assess the long-term safety and efficacy of AgNPs for inflammatory conditions.

## 4. Conclusion

In conclusion, AgNPs exhibit promising anti-inflammatory activity, making them a subject of interest in biomedical research. Their potential to modulate inflammatory responses, inhibit proinflammatory mediators and pathways, and promote wound healing suggests a therapeutic potential in managing various inflammatory conditions. Additionally, AgNPs antimicrobial properties can directly contribute to reducing inflammation by controlling associated infections. However, the translation of AgNPs into clinical applications requires thorough investigation into their safety profile, including potential toxicity and environmental impact. Further research is essential to elucidate the precise mechanisms of action, optimize dosing regimens, and evaluate long-term efficacy in treating inflammatory disorders. Biogenic AgNPs contribute to the treatment of resistant infections or chronic inflammation. With continued study and careful consideration of safety concerns, AgNPs may offer novel therapeutic avenues for addressing inflammation-related diseases in the future. Furthermore, it will be very important to study the toxicity and bioavailability of silver nanoparticles. The smaller the nanoparticles the more they must be bioavailable, enhance distribution, but increase their toxicity due to their larger surface area to volume ratio.

## Figures and Tables

**Table 1:** Tested products.

Batch	Treated groups	Acronyms	Doses
1	Normal saline	Control	10 ml/kg
2	Acetyl salicylic acid	ASA	10 mg/kg
3	Aqueous extract *Aphania senegalensis*	Extract	50 mg/kg
			100 mg/kg
			200 mg/kg
4	Silver nanoparticles	AgNPs	3 mg/kg
			10 mg/kg
			100/Kg

## Data Availability

The data used to support the findings of this study are available from the corresponding author upon request, and these data are archived on the platforms of the institutions Cheikh Anta Diop University and Institut de Chimie des Matériaux de Paris-Est (ICMPE).
